# Exercise-induced brain changes in cannabis use disorder: a longitudinal MRI study of a 12-week supervised HIIT program

**DOI:** 10.1038/s41380-026-03720-2

**Published:** 2026-07-02

**Authors:** Suzan Maleki, Karyn Richardson, Sam Hughes, Edouard Kayayan, Warda Syeda, James P. Coxon, Nadia Solowij, Dan I. Lubman, Valentina Lorenzetti, Karen Caeyenberghs, Rebecca A. Segrave, Chao Suo, Murat Yücel

**Affiliations:** 1 BrainPark, Turner Institute for Brain and Mental Health, School of Psychological Sciences and Monash Biomedical Imaging Facility, Monash University, Clayton, VIC Australia; 2 Melbourne Brain Centre Imaging Unit, Department of Radiology, The University of Melbourne, Parkville, VIC Australia; 3 Movement and Exercise Neuroscience Laboratory, School of Psychological Sciences, Clayton, VIC Australia; 4 School of Psychology, University of Wollongong, Wollongong, NSW Australia; 5 Turning Point, Eastern Health and Monash Addiction Research Centre, Eastern Health Clinical School, Monash University, Clayton, VIC Australia; 6 Healthy Brain and Mind Research Centre, School of Behavioral and Health Sciences, Faculty of Health Sciences, Australian Catholic University, Fitzroy, VIC Australia; 7 Cognitive Neuroscience Unit, School of Psychology, Deakin University, Burwood, VIC Australia; 8 QIMR Berghofer Medical Research Institute, Herston, QLD Australia; 9 School of Psychology, the University of Queensland, St Lucia, QLD Australia

**Keywords:** Addiction, Neuroscience

## Abstract

**Objective:**

Cannabis use disorder (CUD) is highly relapsing and has been associated with structural brain alterations (e.g., white matter and cortical thickness) in pathways and regions critical for a healthy brain. The development of non-abstinence-based interventions is essential for restoring structural alterations in CUD. Physical exercise, particularly aerobic exercise, may promote neuroplastic changes in brain structure.

**Methods:**

In this novel randomised, single-blind, comparator-controlled trial, we recruited 59 individuals with CUD (76% classified with severe CUD; median age = 25 years, 22% female, without requiring abstinence during intervention). They were randomly allocated to receive 12-weeks of 45 min, three times/week of either: (i) *High Intensity Interval Training* (HIIT) aimed to achieve 80% of participants’ maximum heart rate (above the lactate release threshold); or (ii) *active control Strength and Resistance training* (S&R). The main outcome was brain structural organisation (fractional anisotropy and cortical thickness) measured via advanced diffusion and anatomical MRI scans conducted before and after 12 weeks.

**Results:**

The HIIT group showed significant increases in FA in the left uncinate fasciculus (*p* = 0.012) and cortical thickness in the right pars opercularis of the inferior frontal gyrus (*p* = 0.016), revealed by time-by-group interaction. These exercise-induced changes in white matter and cortical thickness significantly correlated with total hours spent with heart rates > 80% (*r* = 0.32, *p* = 0.027 and *r* = 0.41, *p* = 0.001) and the time spent above the lactate release threshold (*r* = 0.27, *p* = 0.061 and *r* = 0.40, *p* = 0.004).

**Conclusion:**

Overall, a 12-week HIIT exercise intervention, without requiring abstinence from cannabis consumption, can enhance brain plasticity.

## Introduction

According to the world drug report, over 280 million people aged 15–64 use cannabis, marking a 26% increase in the past decade and entrenching its position as the most widely consumed illicit substance globally. Cannabis use contributes significantly to the global health burden, with cannabis use disorder (CUD) accounting for approximately 8.5 years of disability-adjusted life lost per 100,000 individuals [1]. The expansion of the cannabis industry and an increase in liberal cannabis policies have paralleled diminishing concerns about the health implications of CUD, while contributing to the rise of new consumption methods (e.g., vaping, edibles) [2]. Worryingly, CUD can be associated with ill mental health (e.g., depression, anxiety), poorer cognitive performance (e.g., disinhibition), and lower quality of life [3].

Further, CUD has been associated with changes in brain structure (e.g., reduced grey matter volume and/or cortical thickness, altered structural connectivity) as demonstrated by structural magnetic resonance imaging (MRI) and diffusion-weighted imaging (DWI) [4]. For example, CUD versus control groups have shown lower volumes in orbitofrontal, hippocampal and medial temporal regions implicated in disinhibition stress, learning and memory controls [5]. Meanwhile, DWI studies revealed widespread white matter (WM) alterations throughout the brain, including the superior longitudinal fasciculus, uncinate fasciculus, corpus callosum, cingulate, and cerebellar peduncles [6, 7]. Overall, there is consistent evidence that CUD is associated with structural brain changes; however, it is unclear whether these changes can be restored.

Emerging evidence suggests that CUD-related brain changes can be restored with abstinence from cannabis [8]. However, abstinence can be difficult to achieve; with ~19% of individuals who experience a CUD reporting unsuccessful attempts to reduce or quit using cannabis, and of these 61% report recurring quit attempts over a 2–3 month period [9]. Effective approaches other than abstinence is required to improve functioning and brain health in CUD. One promising intervention is physical exercise, which offers significant health benefits, including reducing cardiovascular disease and cancer, and improving cognitive performance and mental health [10].

Physical exercise promotes the release of neurotrophic factors and growth hormones (e.g., brain-derived neurotrophic factor, vascular endothelial growth factor, and insulin-like growth factor), which can lead to neuroplastic changes in the brain structure [11]. Interestingly, structural neuroimaging evidence demonstrates that exercise can enhance or restore brain structure in both normative and clinical samples [12, 13]: increased GM volume in the hippocampus, frontal, and parietal regions [13, 14]; increased cortical thickness [15]; improved WM integrity, in the corpus callosum, uncinate fasciculus, and cingulum; and reduced WM hyperintensities according to our previous systemic review [16].

However, variations in exercise regimens and their implementation result in differential physiological and brain outcomes. Firstly, most studies have used moderate continuous exercise (MCE), while the literature suggests that High-Intensity Interval Training (HIIT) outperforms MCE, with HIIT yielding higher and more sustained physiological benefits regarding cardiovascular fitness, lactate levels, and executive functions [17]. A review of HIIT studies in disease (e.g., cardiovascular, psychiatric disorders) has not examined its effect on brain structure [18]. Secondly, previous studies often did not emphasise on structured protocols, sufficient supervision and tailored approaches to maximise engagement and outcomes [19, 20].

This study examines, for the first time (to the author’s knowledge), the effect of an individualised and supervised HIIT exercise protocol on whole brain morphology and improve WM microstructure (at whole brain level and within prior knowledge regions [16]) in CUDs using multimodal structural MRI; with precise physiological assessments (e.g., heart rate monitoring, VO₂-based lactate thresholds). By incorporating accredited exercise physiologists and precisely designed regimens, this study aims to overcome previous limitations and optimise the benefits of exercise. We hypothesise that 12 weeks of tailored HIIT will result in significant brain changes, with exercise quality (e.g., intensity and engagement parameters) driving these effects.

## Methods

### Participants

A total of 59 adults with moderate to severe CUD were recruited. Participants were included if they met the following criteria: 1) moderate to severe CUD as defined by the DSM-V and confirmed with the Mini International Neuropsychiatric Interview, 2) Aged 20–55 years with fluent English, 3) A major history of regular cannabis use defined as ≥ 3 days per week, for an average of four of the past six years (without requiring abstinence). Exclusion Criteria: 1) Significant medical conditions including head injuries or neurological disorder, 2) Lifetime history of psychiatric illness including major depressive disorder, obsessive-compulsive disorder, bipolar disorder, psychosis, autism spectrum, and/or post-traumatic disorder), 3) Experiencing active or unstable use of psychotropic medications, 4) Not eligible for MRI imaging (i.e., claustrophobia, metal implants), 5) Current unstable or chronic medical illness (i.e., cardiovascular disease, musculoskeletal injury or chronic pain) that would prevent safe participation in VO2 max fitness assessments and/or physical exercise, 6) Participation in shift work within the last six months, 7) Completion of five or more HIIT exercises during the past six months, 8) Regular use of illicit drugs other than cannabis, 9) Pregnancy or lactation.

This longitudinal intervention study has been approved by the Monash University Human Research Ethics Committee (Ethics ID #12563, clinical trial registration ID: NCT04902092, and written informed consent was obtained prior to participation). For a detailed study protocol description, see the supplementary material and previous paper [21].

### Intervention

#### Design and randomisation

In this randomised, comparator-controlled, single-blind, parallel trial, 59 participants with CUD were randomised to receive 12 weeks of HIIT or S&R training (comparator control) on a 1:1 ratio using a block randomisation generator. These variables stratified randomisation to ensure that certain age, gender, and cannabis dependency do not impact findings. Participants were blind to the potential superiority of HIIT vs R&S but not to the exercise condition.

#### Exercise conditions

Both exercise conditions required participants to attend 45-min exercise sessions, 3 times per week for 12 weeks in BrainPark facility located at Monash Biomedical Imaging lab, Melbourne (see the supplementary for a schematic overview of the RCT). All intervention sessions were conducted and supervised by an accredited exercise physiologist. All exercise sessions started with a short warmup (i.e., 5–10 min) and ended with a cooldown. Participants were equipped with a Polar heart rate monitor to enable the exercise physiologist to continuously track and modify the exercise intensity ensuring it aligned with the specific parameters of their intervention condition, in real-time. To facilitate engagement across the 12-week period, all trial staff were trained in health behaviour change methodology by Health Change Associates Ltd. During baseline assessments, participants’ knowledge of exercise benefits, their readiness, and confidence to engage in the intervention were assessed. Barriers and facilitators to engagement were also identified and discussed. Throughout the trial, research officers and exercise physiologists used this information to employ motivational strategies such as psychoeducation, motivational interviewing, encouragement, planning, and problem-solving.

#### HIIT intervention

The HIIT intervention included six HIIT protocols with a combination of exercises using stationary devices (Concept2 rower, bikes, and skiergs), boxing and body weight (see supplementary material for details of HIIT protocols). Participants completed six cycles of each protocol throughout the 12-week intervention. Each HIIT protocol involved alternating periods of high-intensity exercise intervals, above 80% of their maximum heart rate (HR Max), lasting between 1 to 4 min, accompanied by rest periods of equal or shorter length. In total, each session included a cumulative 12 min of high-intensity exercise during which the exercise physiologist aimed to ensure participants maintained their exercise intensity above 80% HR max. Exercise intensity was personalised by prescribed heart rate zones corresponding to each participant’s lactate threshold. For example, for one participant 150–170 beats per minute may correspond to 80–90% effort. This approach increased the likelihood of achieving a high-lactate state.

#### S&R intervention

Each strength and resistance session consisted of 2–3 sets with 5–12 repetitions of weighted movements using dumbbells, barbells, bodyweight, and TRX to target major body muscles. The weighted movements of each session were rotated among three different variations: upper and core muscle groups, lower and core, and a combination of all muscle groups (mixed). Participants started the program with a three-week introductory phase consisting exclusively of mixed sessions. From the fourth week onward, they alternated among the three S&R variations on a weekly rotation. The heart rate target was set below 80% HR Max to reduce the chance of entering a high-lactate state. Customised programs were tailored to each participant based on the outlined criteria, with progressive overload principles individually applied under the guidance of exercise physiologists.

#### Exercise engagement measurements (HR and Lactate exposure)

The heart rate (HR) data across 12 weeks (36 sessions) were used to estimate the total duration for which participants exercised above 80% HR Max and beyond their lactate threshold (which was customised for each individual during baseline assessment). Specifically, the lactate threshold is defined as the equilibrium point at which blood lactate production and clearance are balanced; as such, surpassing this threshold leads to an increased accumulation of lactate in the blood [22]. The total hours across the 12 weeks of intervention, spent above 80% of maximum HR, as well as the lactate-releasing phase, was computed.

### Outcome measures

#### MRI protocol

Participants completed their MRI scanning session on a 3 T Siemens Skyra at Monash Biomedical Imaging, Monash University. Anatomical T1-weighted images were acquired using a Magnetization Prepared Rapid Acquisition Gradient-Echo (MPRAGE) sequence with parameters: repetition time (TR) = 2300 ms, echo time (TE) = 2.07 ms, 192 slices, 1 mm^3^ isotropic, field of view = 256 mm by 256 mm; Diffusion MRI sequence consisted of 60-diffusion-encoding gradients conducted with TR = 8800 ms, TE = 110 ms, voxel size = 2.5 mm^3^, right to left phase encoding direction. Each diffusion scan obtained 67 volumes (60 volumes with b = 3000 s/mm^2^ and 7 interleaved b0 volumes). Additionally, opposite phase encoding direction with b = 0 was collected for distortion correction.

#### MRI preprocessing

DWI of both timepoints were processed using MRtrix3 and FSL software [23–25]. Raw diffusion images were denoised and corrected for head motion, eddy current distortions, and bias filed inhomogeneities. For each time point, brain masks were generated from the anatomical T1-weighted images [26] and linearly coregistered to diffusion space using FLIRT in FSL [27]. Individual fractional anisotropy (FA) maps were computed using FMRIB Diffusion Toolbox, and then nonlinearly transformed to the target image (FMRIB58_FA) and affined transformed to MNI152 space using standard Tract Based Spatial Statistics (TBSS) pipeline [28]. For whole brain level comparison, individual longitudinal FA changes were computed by subtracting baseline FA from their follow-up FA image.

Anatomical T1-weighted images of both timepoints were processed using the standard FreeSurfer Longitudinal pipeline (FreeSurfer version 6.0). In brief, each scan was independently (cross-sectionally) processed, then the processed scans were coregistered and averaged to create individual unbiased templates, followed by longitudinal processing according to this unbiased template [29]. To check the quality of longitudinal coregisteration, the quality of individual templates and across-time registration were inspected slice by slice. Further, ENIGMA quality control procedures were applied in FreeSurfer cortical measures to ensure data accuracy and reliability (see supplementary). As standard longitudinal FreeSurfer processing [29], the percent change of cortical thickness (mm/year) with respect to baseline (PC1) was computed for each hemisphere individually followed by smoothing at 10 mm.

$${rate}({change}\,\,^{mm}/_{year})=\frac{{FU}\,{cortical}\,{thickness}-{BL}\,{cortical}\,{thickness}\,}{{FU}\,{time}-{BL}\,{time}}$$

$${PC}1=\frac{{rate}}{{BL}\,{cortical}\,{thickness}}$$

#### Cannabis use measures

The total amount of cannabis consumed during the four weeks preceding both baseline and endpoint assessments and across the intervention period was evaluated using the Time Follow Back Procedure (TLFB) [30].

#### Cognitive measures

Cognitive performance was measured with the CANTAB using Paired Associate Learning test (using FAMS and TEA scores) and the Rey Auditory Verbal Learning Test (using total learning and recognition scores).

### Statistical analysis

Longitudinal FA maps were generated by subtracting baseline FA from their corresponding follow-up maps individually. To determine the whole brain group by time differences, a non-parametric permutation approach (*N* = 5000) was performed for both contrasts (i.e., HIIT > S&R, HIIT < S&R) using FSL while controlling for age, sex, and total intracranial volume [31]. For ROI analysis: the mean of longitudinal FA changes (Follow up – Baseline) were extracted from four tracts of interest from the JHU (John Hopkins University) atlas, which have been shown to change following exercise [16], including the corpus callosum, cingulum, uncinate fasciculus, and internal capsule. Two-sample t-tests were used after variance similarity was checked by Levene’s tests, followed by a rigorous Bonferroni correction.

To investigate the whole brain group by time differences in cortical thickness, a longitudinal GLM was performed with a cluster-wise multiple comparison at a significance threshold of *p* < 0.01 (non-parametric, 1000 permutations simulations) while controlling for age, gender, total intracranial volume, and years of education.

Finally, associations between regional FA values, cortical thickness values in the GM regions, which showed significant group differences, cannabis use, and exercise engagement parameters (HR and lactate) were examined using Pearson correlations while controlling for age, gender, and years of education in SPSS (*p* < 0.05). Data met assumptions for the statistical test applied.

## Results

### Descriptive

Fifty-nine participants with CUD, aged 20–53 (median age: 25 years, 22% female), completed baseline assessments. According to the MINI interview, 76% of participants had severe CUD with a mean age of onset at 19.52 (± 4.72) and an average of 7.48 (± 6.49) years of regular cannabis use. Eleven participants did not complete the intervention (due to conflicting life demands (*n* = 6), the initiation of excluded medication (*n* = 1), or the COVID-19 pandemic (*n* = 3)). One participant was excluded due to poor-quality MRI data (motion artifact). Overall, 47 participants (81%) completed the 12-week intervention. See the supplementary for a schematic overview of the RCT protocol.

### Longitudinal engagement with exercise

**Fig. 1:**
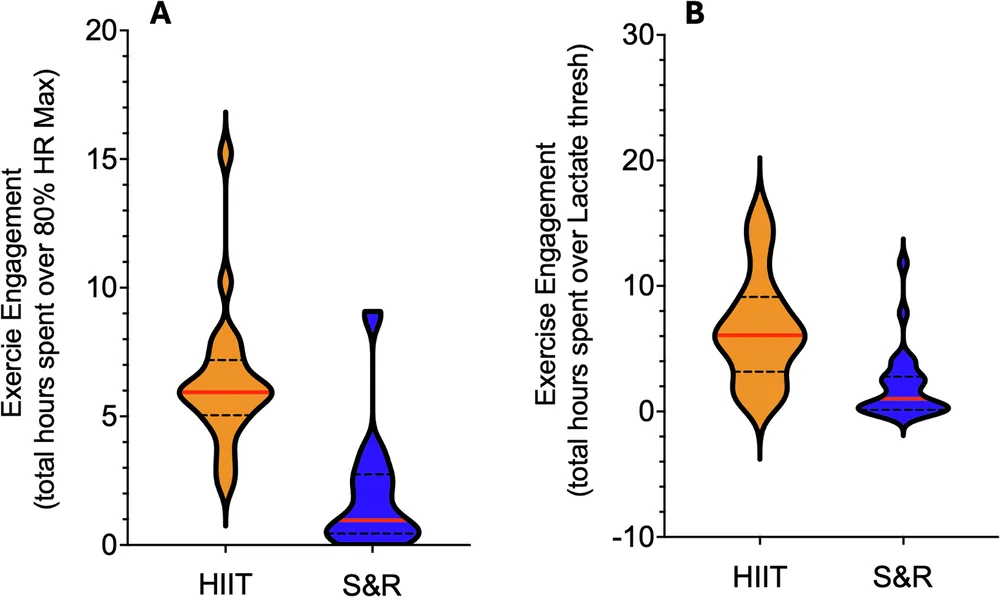
Significant group-by-time differences in exercise engagement parameters. During the 12 weeks intervention, the total hours that Individuals in the HIIT group (*N* = 22) exercised above 80% HR Max (*p* < 0.0001, **Panel A**) and over the lactate threshold (*p* < 0.0001, **Panel B**) was significantly higher than the S&R group (*N* = 25). Dashed lines indicate 95% CIs.

Participants in the HIIT group spent significantly more time exercising above 80% of their maximum heart rate (*p* < 0.0001) and above their lactate threshold (*p* < 0.0001) compared to the S&R group (Fig. 1). Additionally, time spent exercising above 80% HR Max and lactate threshold showed a positive correlation (*r* = +0.75, *p* = 0.0001), confirming consistency between these engagement measures.

### Changes in cannabis use

There were no significant longitudinal changes in levels of cannabis use between the HIIT vs S&R group.

### Group by time interaction effect on brain plasticity

#### White matter organisation

**Fig. 2:**
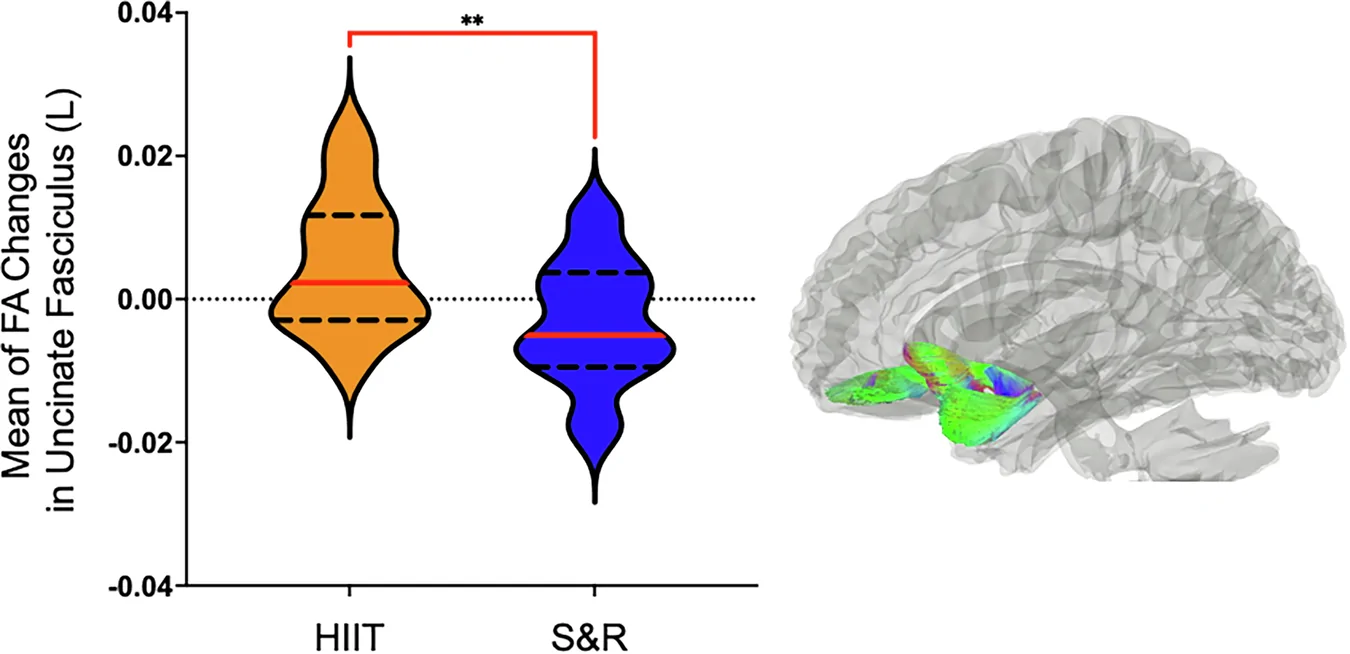
The left violin plot illustrates the significant increases in FA values after 12 weeks HIIT in the left uncinate fasciculus tract compared to the S&R group, (**p < 0.01, multiple comparison corrected *p* = 0.012). This WM tract is shown on the right panel for illustrative purposes only. Dashed lines indicate 95% CIs.

The whole brain analysis using TBSS revealed no significant group by time effect on FA using threshold-free cluster-wise multiple comparison correction. The ROI analysis revealed an increase in FA changes in the left uncinate fasciculus following HIIT compared to the S&R group (*p* = 0.004), which remained significant after Bonferroni correction (*p* = 0.012), as shown in Fig. 2.

#### Cortical thickness

**Fig. 3:**
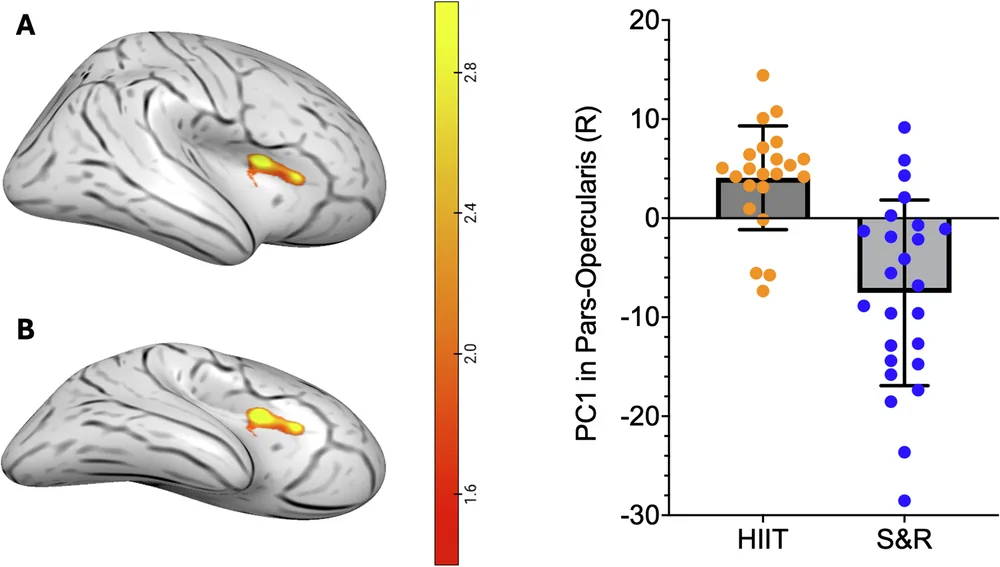
Significant increase of cortical thickness (PC1) in the right inferior frontal gyrus (i.e.,-pars-opercularis) in the HIIT group (*N* = 22) compared to the S&R group (*N* = 25) after the 12-week of intervention. **A** illustrates the right hemisphere and (**B**) depicts the 45 degrees tilted view. The cluster size is 370 mm^2^ with cluster-wise multiple comparison corrected *p*-value = 0.016 (MNI coordinates of X = 38.2, Y = 9.0, Z = 12.2). The right panel illustrates the PC1 changes of individuals from each group (separated by colour) is presented. Error bars indicate standard deviation.

Our whole-brain analysis revealed significant group-by-time effects on the cortical thickness values in the right pars-opercularis region of the inferior frontal gyrus (IFG) using FDR correction. Compared to the S&R group, cortical thickness values in the right pars opercularis of the inferior frontal gyrus increased in the participants undergoing HIIT intervention, Fig. 3 (*PC1*: *p* = 0.016, cluster size = 369.21 *mm*^2^).

### Correlation analysis

**Fig. 4:**
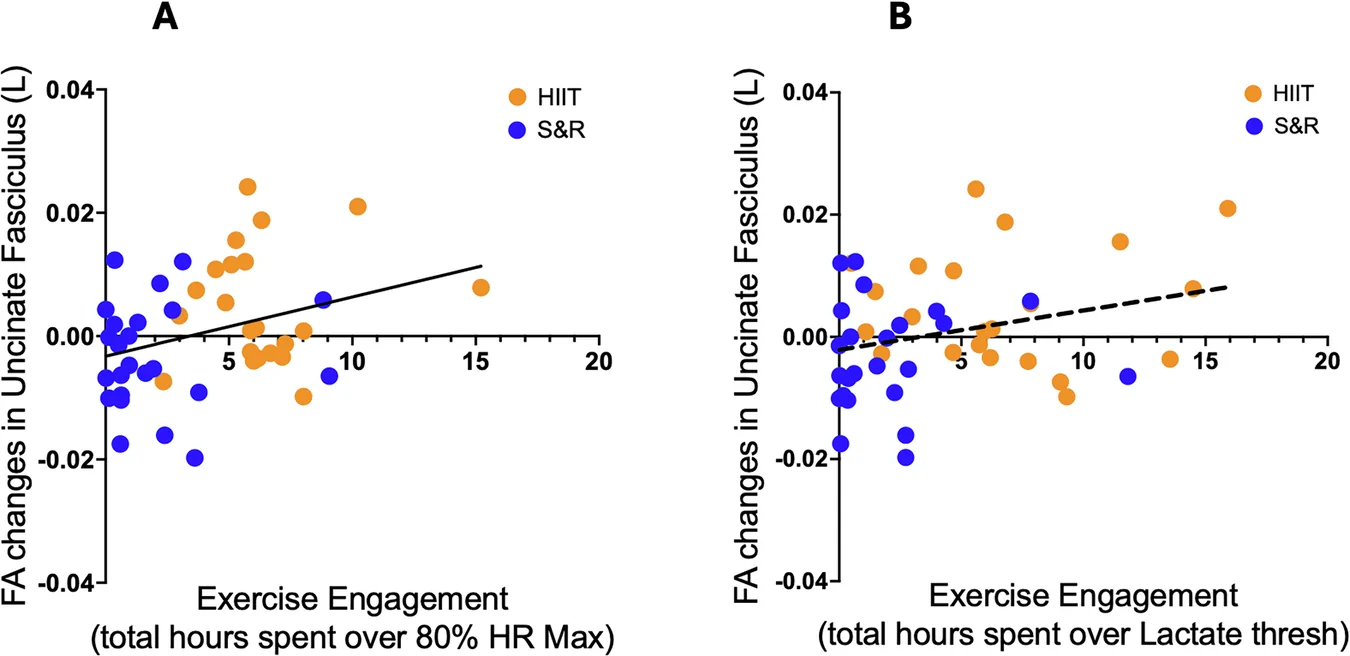
Positive correlation between longitudinal FA changes in the left-uncinate fasciculus region and exercise engagement variables. **A**: Significant association between regional FA changes and total hours of heart rate beating above 80% HR Max (r = +0.32, *p* = 0.027). **B**: Positive trend between regional FA increase and total hours of training spent above lactate release threshold (r = +0.27, *p* = 0.061).

Given the significant group-by-time differences observed in exercise engagement parameters (e.g., HR Max and lactate) and brain structure measurements, we conducted a correlation analysis to examine potential associations between these outcomes. We observed positive significant correlations between the longitudinal FA changes in the left uncinate fasciculus and the exercise engagement measurements across two groups: for total hours of 80% HR Max *r* = +0.32, *p* = 0.027 (Fig. 4A); and a trend for total hours spent above lactate threshold *r* = +0.27, *p* = 0.06 (Fig. 4B). In other words, participants who spent time above the lactate threshold showed more FA changes in the left uncinate fasciculus.

**Fig. 5:**
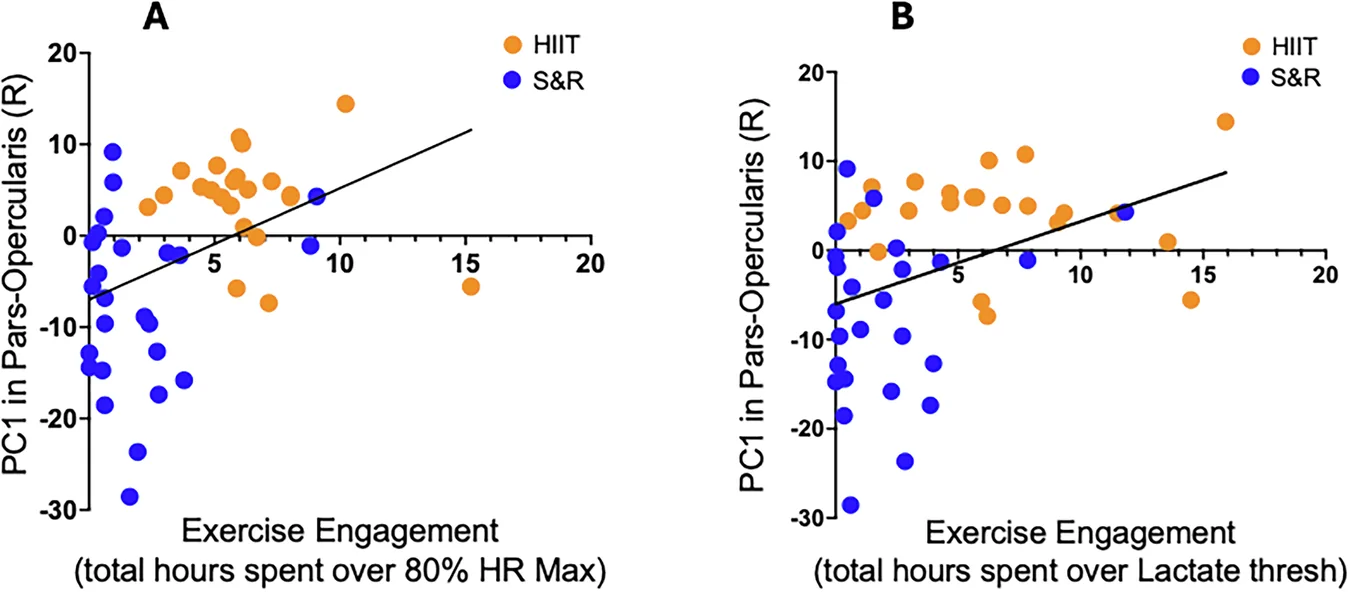
Significant positive association between cortical thickness changes (PC1) in right-Parsopercularis regions and exercise engagement parameters (r = +0.47, *p* = 0.001). **A**: Significant association between regional cortical thickness and total hours of heartrate being above 80% of individuals’ maximum heart rate. **B**: Significant positive correlation between cortical thickness changes and total hours within lactate release phase (r = +0.41, *p* = 0.004).

Changes in PC1 of the right IFG (pars-opercularis) region were also positively associated with total hours exercising above 80% HR Max *r* = +0.47, *p* = 0.001 (Fig. 5A) and lactate threshold *r* = +0.41, *p* = 0.004 (Fig. 5B).

#### Exploratory

FA increases in the uncinate fasciculus were positively associated with cortical thickness increases in the IFG (pars-opercularis) region (*r* = +0.36, *p* = 0.012). Further, a negative trend level association with changes in cortical thickness and total cannabis consumption before and after the intervention (*r* = −0.25, *p* = 0.08), indicating that reduced use was associated with greater increases in cortical thickness. No other significant correlations were observed.

### Changes in cognition

There were no significant changes in cognitive performance following HIIT relative to S&R training group. No associations emerged between WM metrics and cognitive measures.

## Discussion

This randomised clinical trial evaluated the efficacy of 12 weeks of HIIT aerobic exercise on brain structure in individuals with CUD. While no significant whole-brain structural connectivity changes were observed, HIIT increased regional WM integrity in the left uncinate fasciculus compared to S&R training. However, cortical thickness increased at the whole-brain level following HIIT in the right inferior frontal gyrus (pars opercularis) compared to S&R training. Throughout the trial, these structural brain changes were positively associated with exercise engagement parameters (total hours spent above 80% HR Max, lactate threshold). Overall, HIIT can enhance distinct metrics of brain structure in CUD, without requiring abstinence.

The FA changes observed in the uncinate fasciculus align with prior evidence [16]. This critical WM pathway connects key regions of the addiction neurocircuitry (e.g., orbitofrontal cortex) with medial temporal areas (e.g., amygdala and hippocampus) implicated in emotion regulation and impaired in CUD [6, 32]. Literature suggests that FA changes respond to new activities, supporting dose-dependent effects [33, 34]. The significant association between WM increase and time spent above 80% HR Max suggests exercise intensity (i.e., HIIT vs S&R) drives improvements in WM integrity [35]. However, a caveat to the above is that changes in WM could be also related to broader factors beyond exercise, such as changes in participants level of motivation or stress as well as expectancy effects. Additional measurements of these factors are encouraged in future studies.

The 12 weeks of HIIT increased cortical thickness in the right IFG (i.e., pars-opercularis), a region critical for impulse control and response inhibition [36]. This aligns with prior evidence of exercise-induced increases in cortical thickness. For instance, Suo and colleagues (2016) reported increased posterior cingulate thickness following six months of exercise in older adults with cognitive impairment [15]. Also, Stern and colleagues (2019) observed increases in the frontal region in healthy individuals after six months of aerobic training [37]. This finding suggests that exercise-induced neuroplasticity strengthens this frontal region, which is commonly affected in individuals with CUD. While these findings differ regionally, likely attributable to variations in exercise types, engagement levels, and demographics such as age, the overall evidence highlights exercise’s role in promoting MR-detectable brain plasticity. Similar to WM changes, the increase in cortical thickness was also positively correlated with total time spent above 80% HR Max and lactate threshold. Although direct studies linking physiological responses to brain structure are limited, research shows exercise intensity correlates with total GM, WM and regional GM volumes in the frontal and parietal lobes [13, 38].

Further research is warranted, but the significant correlation between WM integrity and cortical thickness suggests cross-modal alterations may reflect concurrent neural adaptations. Anatomically, the proximity of this WM tract to the IFG [39], though not directly to the pars opercularis, supports this association.

Though the exact neurobiological mechanism of exercise-induced structural change remains unclear, many studies suggest exercise can increase Brain-Derived Neurotrophic Factor (BDNF) and Vascular Endothelial Growth Factor (VEFG) expression [40, 41], which is a key driver of brain plasticity. As we did not measure these factors, it is difficult to provide direct evidence on the mechanism at this level. Apart from neurobiological mechanisms, hormonal and physiological responses can be influenced by exercise and the parameters of exercise, such as frequency, duration and intensity [42]. As such, we carefully integrated these exercise variables into this intervention, aligning with previous studies emphasising the necessity of achieving specific exercise parameters to stimulate brain plasticity and enhance neural health [38, 42]. Notably, the observed brain structure group differences (increased WM integrity and GM thickness) were associated with exercise regimen parameters (i.e., 80% HR Max and lactate threshold), highlighting their role as key drivers of the observed effects and validating the tailored protocol’s design for promoting brain plasticity. Further, nearly 80% of participants completed the 12-week intervention, attending an average of 80% of sessions. Motivational strategies, including supervised training sessions by qualified and highly trained staff, were vital in promoting accountability, ensuring safe and correct exercise execution, boosting encouragement, and fostering relationships with exercise physiologists and peers. These findings align with a systematic review showing exercise programs incorporating individualised approaches and social support achieve better clinical outcomes and higher retention rates [43].

Several considerations should be noted when interpreting the present findings. Participants were not required to abstain from cannabis use during the intervention; an intentional design choice aimed at enhancing ecological validity by more closely reflecting real-world clinical contexts. However, this necessarily limits the generalisability of the results to abstinent individuals with CUD. The absence of detectable whole-brain structural changes may therefore reflect ongoing cannabis-related neurobiological disruptions, including intracellular THC accumulation, altered cannabinoid receptor signalling, and vascular or dendritic impairments, all of which may blunt exercise-induced neuroplastic adaptations and associated cognitive benefits. Further, the cognitive tasks were more targeted in the memory domain. Based on the current observation of the exercise induced brain changes, tasks that targeting these region and function domains (impulse control, inhibition and emotion regulation) would be recommended for similar study in future (e.g. Stop-Signal Task or Go/No-Go Task). In addition, both HIIT and strength-and-resistance training produced measurable benefits, which may have reduced MRI-detectable differences between intervention arms. Future studies incorporating an inactive control group and abstinence-stratified designs will be important for disentangling exercise-specific effects from ongoing substance-related influences.

This is the first longitudinal study to demonstrate that 12 weeks of HIIT exercise can enhance brain plasticity in individuals with CUD without abstinence, using advanced multimodal MRI and gold-standard protocols for design, delivery, and assessment regarding exercise. These findings reveal that HIIT increased WM integrity and cortical thickness in regions involved in response inhibition control. These exercise-induced brain changes were strongly linked to exercise engagement, emphasising the potential of personalised exercise prescriptions to positively influence brain health in individuals with CUD. From a translational perspective, these findings support the feasibility of implementing HIIT-based exercise as an adjunctive intervention for individuals with CUD in real-world settings. Community-based delivery models—such as structured HIIT programs embedded within youth recreation centres, fitness facilities, or community mental health services—may offer accessible, scalable, and non-stigmatising entry points for engagement, particularly when delivered in partnership with schools, community organisations, and healthcare providers. In parallel, digitally delivered or hybrid exercise models incorporating app-based guidance, telehealth supervision, and wearable monitoring may further enhance reach, flexibility, and adherence by reducing barriers related to transport, scheduling, and stigma. Embedding personalised exercise prescriptions within established therapeutic frameworks, such as cognitive–behavioural therapy or motivational interviewing, may maximise both neurobiological and psychosocial benefits, supporting integration into existing clinical and public health pathways.

## Supplementary information

Supplementray material 1

Supplementary material 2 eMethods
