# Supplementary material for: Exercise-induced brain changes in cannabis use disorder: a longitudinal MRI study of a 12-week supervised HIIT program
Source: Mol Psychiatry. 2026 Jul 2;31(11):6510–6. doi: 10.1038/s41380-026-03720-2 (PMC13668721; doi:10.1038/s41380-026-03720-2)
Supplement: Supplementary file 1 [file 41380_2026_3720_MOESM1_ESM.docx]

# Supplementary material

## 1. The schematic overview of the randomised control trial

## 2. Quality Control steps on FreeSurfer cortical outcomes

The updated ENIGMA quality control protocol (April 2017) was applied to the FreeSurfer-derived cortical measures at both baseline and follow-up timepoints. This comprehensive protocol, which includes script packages, quality control guidelines, and relevant templates, was fully implemented to ensure data integrity and consistency across all scans. All steps and scripts are available online: <https://enigma.ini.usc.edu/protocols/imaging-protocols/>.

Below is a concise summary of the quality control procedures implemented:

**Step 1: Data Extraction and Organisation**

Cortical measures were extracted using FreeSurfer and organised within the designated FreeSurfer directory. Using the ENIGMA scripts, surface area and cortical thickness values were computed for each region of interest (ROI) for all participants at both baseline and follow-up timepoints.

**Step 2: Quality Control of Cortical Surface Segmentations**

Three main sub-steps were applied to assess the quality of cortical surface segmentation outputs:

A. Outlier Detection: Subjects with cortical thickness and surface area values significantly deviating from the group distribution were identified using the ENIGMA outlier detection script in R.

B. Internal Surface Method: Cortical surface segmentations were overlaid onto individual anatomical scans, and internal slices were compiled into an HTML-based viewer using the ENIGMA MATLAB function for efficient inspection.

C. External Surface Method: External views of the cortical segmentations were generated from multiple angles and displayed in a webpage for visual assessment.

**Step 3: Summary Statistics and Visualisations**

Summary statistics and histogram plots were generated using the ENIGMA R scripts further to evaluate the distribution and consistency of cortical measures.

**Step 4: Visual Inspection and Rating**

Following ENIGMA’s internal and external quality control guidelines, outputs were visually inspected and rated (e.g., pass, moderate, fail), with hemisphere-specific annotations. Ratings were recorded in the ENIGMA-provided Excel template. None of the participants were flagged with critical segmentation errors or significant outliers. Subsequently, all subjects were included for analyses based on these ratings.
