# Supplementary material for: Exercise-induced brain changes in cannabis use disorder: a longitudinal MRI study of a 12-week supervised HIIT program
Source: Mol Psychiatry. 2026 Jul 2;31(11):6510–6. doi: 10.1038/s41380-026-03720-2 (PMC13668721; doi:10.1038/s41380-026-03720-2)
Supplement: Supplementary file 2 [file 41380_2026_3720_MOESM2_ESM.docx]

**Brain exercise and Addiction Trial Methods**

**eMethods**

eFigure 1: HIIT Protocol A: Mixed Functional Exercises

eFigure 1: HIIT Protocol B: Mixed Functional Exercises

eFigure 1: HIIT Protocol C: Mixed Functional Exercises

eFigure 1: HIIT Protocol D: Mixed Functional Exercises

eFigure 1: HIIT Protocol E: Mixed Functional Exercises

eFigure 1: HIIT Protocol F: Mixed Functional Exercises

eTable 1: Strength and Resistance Protocol: Mixed body groups template

eTable 2: Strength and Resistance Protocol: Upper and lower muscle groups template

eTable 3: Strength and Resistance Protocol: Upper and lower muscle groups template

eFigure 7: Hippocampal integrity – reprint from Yücel et al. 2016

**eResults**

eTable 4. *Demographic and Baseline Characteristics*

eTable 5. Exercise engagement between participant who did, or did not have at least one exercise session via video-call.

**eReferences**

This supplemental material has been provided by the authors to give readers additional information about their work. 2

**eMethods**

Few studies to date have tailored exercise prescription to target precise physiological mechanisms (i.e., low vs high lactate exposure) or comprehensively assessed the components of hippocampal integrity and its functional consequences, using multimodal techniques. Engaging people in the level and frequency of exercise required to influence neuroplasticity, or active comparators which are often less appealing to participants (e.g., walking or stretching programs), is also notoriously difficult, and more so for people living with a substance use disorder^1,2^. Adequate engagement is necessary to elucidate true findings and therefore the appropriateness of exercise for particular outcomes and cohorts. Behavioural science indicates that group-based exercise interventions, supervised by an exercise professional who can provide personalised motivational support, are most effective in encouraging exercise participation^3–5^. As such, the current trial used a behaviourally informed approach that brings together gold-standard principles in exercise science, neuroscience, and behaviour change to determine the impacts of regular exercise in CUD. Exercise intensity was personalised to each participants’ lactate threshold based on cardiopulmonary exercise testing and real-time exertion monitoring during sessions, such that HIIT was prescribed and monitored at above lactate threshold and S&R below it. The intervention was delivered by Accredited Exercise Physiologists (AEPs) who also provided motivational support based on each participants’ unique circumstances (i.e., specific barriers and facilitators to exercise) to maximise retention, adherence, and intervention fidelity.

This trial was approved by the Monash University Human Research Ethics Committee (Ethics approval number#12563) and was registered on the National Institutes of Health Clinical Trials Registry (NCT04902092). The trial was conducted between January 2019 and December 2022.

***Design, Randomisation, and Blinding***

A randomised, comparator-controlled, parallel group design was employed. Participants were randomised to 12-weeks of HIIT or S&R training on a 1:1 basis at the end of their baseline assessment using a predetermined randomisation schedule constructed with a random number generator. Block randomisation (generated with a random number sequencer by independent researcher) was used to stratify the sample according to CUD severity (substance dependence scale, (SDS)), age, and gender. Participants were blind to the hypothesised superiority of HIIT in enhancing hippocampal integrity but not to exercise condition. Trial investigators and research officers conducting outcome assessments assigned participants to the intervention and were not blind to exercise condition. The researcher conducting analysis of the primary outcome (including manual delineation of hippocampal volume) was blinded to group allocation, and other measures contributing to the primary outcome were generated using computer scripts to ensure consistency and minimise potential bias.

***Participants***

Participants were recruited via advertisements placed throughout the community and on social media platforms (i.e. Twitter, Facebook, Instagram). Eligible participants were adults aged 20 to 55, with moderate to severe CUD, as defined by the DSM-V, and confirmed with the Mini International Neuropsychiatric Interview. Inclusion criteria also included a significant history of cannabis use, defined as three or more days of use per week, for an average of four of the past six years. Exclusion criteria were: current engagement in CUD treatment, presence of a neurological disorder or serious head injury; lifetime history of bipolar disorder, obsessive-compulsive disorder, post-traumatic stress disorder, psychosis or autism spectrum disorder; current, unstable use of psychotropic medication; contraindications for MRI (i.e., metal implants and claustrophobia); current unstable, or chronic medical illness (i.e., cardiovascular disease, chronic pain, musculoskeletal injury), that would preclude safe engagement in CPET and/or regular physical exercise; engaging in shift work in the previous six months; engaging in five or more HIIT sessions within the previous six months; moderate and/or severe substance use disorder other than CUD (as defined by the MINI); pregnancy or lactation.

***Sample size***

Sample size estimates were based on planned comparisons for the primary outcome, hippocampal integrity. Power and sample size software (PASS, NCSS, LLC) was used to estimate the sample size required for a two by two Generalised Estimating Equations design, with exercise condition as the between-subjects variable, and time as the within-subjects variable. Assuming a 1.0 difference in means between groups (with a standard deviation of 2.3, based on hippocampal integrity calculations from previously published data^7^ and that changes in hippocampal integrity would be negligible in the comparator group, a total sample size of 75 participants was required to observe an effect size of 80% power.

***Procedures***

Advertisements directed potential participants to an online Qualtrics survey (Qualtrics, Provo, UT) which assessed likely eligibility based on age, frequency of cannabis consumption, and history of a psychotic illness. Potential participants then proceeded to a telephone screen that assessed all inclusion/exclusion criteria in detail. If eligible, baseline assessments were scheduled. Assessments were conducted in person at Monash University over two three-hour sessions completed on separate days that were no more than seven days apart. At baseline session one, written informed consent was obtained and demographic, cannabis use, mental health, and cognitive measures were completed. At baseline session two, participants underwent MRI and CPET procedures. For endpoint assessments, this order was reversed so that MRI and CPET testing occurred during endpoint session one and all other measures were completed during endpoint session two. HIIT and S&R sessions were conducted at Monash University’s BrainPark gym. The trial was paused between March and November 2020 due to the Coronavirus Disease 2019 (COVID-19) pandemic and associated public health lockdowns in Melbourne. From November 2020 until November 2021, demographic, cannabis use, mental health, and cognitive measures were completed online via a video-call in accordance with the University’s COVID safety protocols. Video-call exercise sessions were also implemented to minimise the disruption from ongoing COVID-19 lockdowns. Online exercise protocols were carefully developed and tested by the trial AEP to ensure fidelity across online and in-person sessions and that online protocols achieved the desired level of exercise intensity from participants. All participants were provided with a Polar HR monitor to wear at home during video based sessions. Where necessary, participants were provided with basic equipment (i.e., exercise mat, resistance bands). Assessment session one and exercise sessions continued to be offered online after COVID-19 safety procedures were lifted. To compensate for their time, participants were reimbursed $50 for each assessment time point.

***Intervention***

Both exercise conditions required participants to attend three 45-minute exercise-sessions per week for 12-weeks. Sessions were delivered in small groups (maximum six participants) or one-on-one. All sessions were delivered by an AEP (SH or EK). Exercise sessions began with a brief warmup and ended with a cool down. Participants wore a Polar H10 heart rate (HR) monitor which allowed the AEP to monitor exercise intensity in real time and adjust the intensity to fit within the parameters of their specified intervention based on personalised LT (see below). To maximise participant engagement, each intervention included prescriptive strategies to increase variety (e.g., a variety of exercise equipment, work-rest time variations (HIIT), a selection of functional exercises (HIIT) and a variety of strength exercises (S&R). See eTable 1 and 2 for full details of the exercise library.

***HIIT***

The HIIT condition consisted of six HIIT protocols (eFigure 1-6) that used a combination of stationary machines (Concept 2 rowers, bikes, and skiergs), boxing, and functional bodyweight exercises. Each HIIT protocol involved alternating bouts of high intensity exercise (>80% HRmax) and rest periods (<80% HRmax). The cumulative amount of high intensity exercise equated to 12-minutes per session. High-intensity intervals ranged from 1-4 minutes in duration with rest periods of equivalent duration or less. During high-intensity bouts, AEPs aimed to ensure that participants were exercising above 80% of their maximum HR, to increase the likelihood of achieving a high-lactate state. If necessary, exercise intensity was titrated during the first three weeks of the intervention based on each participant’s confidence, exercise tolerance, and motor ability. Participants completed six cycles of each protocol over the course of the 12-week intervention. Speed or exercise load was adjusted to ensure target heart rates were achieved. When exercise sessions were conducted via video-call sessions exercises using stationary equipment were replaced with functional body weight exercises.

eFigure 1: HIIT Protocol A: Mixed Functional Exercises

i) Interval and rest times

ii) Audio-visual guide

eFigure 2: HIIT Protocol B: Boxing and Machines

i) Interval and rest times

ii) Audio-visual guide

eFigure S: HIIT Protocol C: Functional Exercises and Machines

i) Interval and rest times

ii) Audio-visual guide

eFigure 4: HIIT Protocol D: Boxing

i) Interval and rest times

ii) Audio-visual guide

eFigure 5: HIIT Protocol E: Functional and machines

i) Interval and rest times

ii) Audio-visual guide

eFigure 6: HIIT Protocol F: Boxing and functional exercises

i) Interval and rest times

ii) Audio-visual guide

***Strength and Resistance Training***

The S&R active control condition targeted large muscle groups and alternated between three focused S&R variations: upper body and core muscle groups, upper and lower body muscle groups, and a combination of all muscle groups (mixed, eTables 1-3). Participants began the program with a three-week progressive ramp of only mixed sessions. From week four onwards participants rotated through each of the three S&R variations every week. In each session, participants completed 2-3 sets, of 5-12 repetitions of loaded movements. The HR target was <80% HRmax, to minimise the likelihood of achieving a high-lactate state. The exercise equipment used included dumbbells, barbells, Total Resistance Exercise Suspension bands, and bodyweight. Tailored programs were designed using the above criteria and the principles of progressive overload were applied on an individual basis at the discretion of the AEPs. For the S&R condition, exercises were progressed by making adjustments to intensity, repetitions, or movement difficulty. When S&R sessions were conducted via video-call participants used their own dumbbells or were provided with resistance bands.

eTable 3: Strength and Resistance Protocol: Mixed body groups template

| **Focus** | **Exercise** | **Sets** | **Reps** | **Weight** | **Image** |
| --- | --- | --- | --- | --- | --- |
|  | | | | | |
| **Lower** | Selected from exercise library | 2-3 | Determined by AEP based on progression | Determined by AEP based on progression |  |
| **Upper** |  |  |  |  |  |
| **Core** |  |  |  |  |  |
|  | | | | | |
| **Lower** | Selected from exercise library | 2-3 | Determined by AEP based on progression | Determined by AEP based on progression |  |
| **Upper** |  |  |  |  |  |
| **Core** |  |  |  |  |  |
|  | | | | | |

eTable 4: Strength and Resistance Protocol: Upper and lower muscle groups template

| **Focus** | **Exercise** | **Sets** | **Reps** | **Weight** | **Image** |
| --- | --- | --- | --- | --- | --- |
|  | | | | | |
| **Upper** | Selected from exercise library | 2-3 | Determined by AEP based on progression | Determined by AEP based on progression |  |
| **Lower** |  |  |  |  |  |
|  | | | | | |
| **Upper** | Selected from exercise library | 2-3 | Determined by AEP based on progression | Determined by AEP based on progression |  |
| **Lower** |  |  |  |  |  |
|  | | | | | |
| **Upper** | Selected from exercise library | 2-3 | Determined by AEP based on progression | Determined by AEP based on progression |  |
| **Lower** |  |  |  |  |  |
|  | | | | | |

eTable 5: Strength and Resistance Protocol: Upper and lower muscle groups template

| **Focus** | **Exercise** | **Sets** | **Reps** | **Weight** | **Image** |
| --- | --- | --- | --- | --- | --- |
|  | | | | | |
| **Core** | Selected from exercise library | 2-3 | Determined by AEP based on progression | Determined by AEP based on progression |  |
| **Lower** |  |  |  |  |  |
|  | | | | | |
| **Core** | Selected from exercise library | 2-3 | Determined by AEP based on progression | Determined by AEP based on progression |  |
| **Lower** |  |  |  |  |  |
|  | | | | | |
| **Core** | Selected from exercise library | 2-3 | Determined by AEP based on progression | Determined by AEP based on progression |  |
| **Lower** |  |  |  |  |  |
|  | | | | | |

***Motivational Strategies***

All trial staff were trained in person centred health behaviour change methodology (Health Change Associates Ltd). Research Officers’ minimum qualification was an Honours degree in Psychology. During baseline assessment, participants’ knowledge about the health benefits of exercise, the importance of achieving these outcomes, and their readiness and confidence to engage in the intervention were evaluated and addressed as necessary. Potential behavioural, emotional, situational, and cognitive barriers and facilitators to engagement were also identified and discussed. Throughout the trial, Research Officers and AEPs used this information to employ motivational and support strategies including psychoeducation, encouragement, planning, and problem solving as required. Participants were also paid $15 for each exercise session they attended.

***Outcome Measures***

***Primary outcome measure***

The primary outcome was left hippocampal integrity which is a composite of three well-validated indices of hippocampal health known to be compromised following long-term exposure to cannabis (eFigure 7): (i) volume, (ii) N-acetylaspartate, and (iii) fractional anisotropy (FA)^7^. Each hippocampal measure was separately normalised into z-scores using the mean and standard deviation of the entire sample’s baseline data. Hippocampal integrity was then calculated as a composite of the three z-scores.

eFigure 7: Hippocampal integrity – reprint from Yücel et al. 2016

Using these methods, a dose-dependent reduction in volume ^8–12^, a decrease in connectivity and axonal fibre integrity^12,13^, and reduced concentrations of n-acetylaspartate (NAA), a marker of neuronal health, have been described in the hippocampi of people with CUD^7^. As a critical region for learning, memory, and emotional processing, the integrity of the hippocampus is functionally related to many of the brain-based symptoms of CUD. For example, neuroimaging studies have demonstrated an association between reduced hippocampal volume and poor verbal learning and memory, and lower activation in hippocampal and prefrontal regions during memory tasks in people who use cannabis^14^. Similarly, extensive research has demonstrated an association between depression and reduced hippocampal volume^15,16^, while altered hippocampal function and connectivity have been linked to anxiety^17^ and apathy^18,19^, respectively.

***MRI Protocol and Procedure***

Each participant underwent a comprehensive imaging protocol on a 3-Tesla Siemens Skyra MRI scanner with a 32-channel head coil at Monash Biomedical Imaging Facility, including T1 weighted structural MRI (T1w), DTI, and MRS. The scanning protocol for each MR modality was as follows: Anatomical T1-weighted images (T1w) were acquired using a Magnetization Prepared Rapid Acquisition Gradient-Echo (MPRAGE) sequence with parameters: repetition time (TR)=2300ms, echo time (TE)=2.07ms, 192 slices, 1mm^3^ isotropic, field of view = 256mm by 256mm; DTI sequence consisted of 60-diffusion-encoding gradients conducted with TR=8800ms, TE=110ms, voxel size=2.5 mm^3^, R>>L phase encoding direction. Each diffusion scan obtained 67 volumes (60 volumes with b=3000 s/ mm2, and 7 interleaved b0 volumes).  Single voxel MRS was conducted at left hippocampus (TR/TE = 2000/30 ms, 256 averages, voxel size = 20x15x30 mm), followed by a non-waster suppression MRS sequence with the same parameter and localization except for 16 averages for water reference. Task based fMRI: The scanning protocol of both the encoding and retrieval phases used the following parameters: TR = 2500ms, TE = 30ms, flip angle = 90°, field of view = 192mm, matrix = 64, voxel size 3mm3, 44 slices without gap, except for 82 volumes for memory encoding (~ 3mins) and 164 volumes for memory retrieving (~5mins).

*Hippocampal Volume*

Hippocampal volume was calculated using FIRST (Functional MRI of the BRAIN (FMRIB)’s Integrated Registration and Segmentation Tool) in FMRIB’s Software Library^20^, which segmented the hippocampus based on T1-weighted images. The method and quality checks were applied based on the standard ENIGMA protocol^21^, and hippocampal volumes were adjusted by total intracranial volume using previously published methods^7^.

*Fractional anisotropy*

Raw DTI data were visually checked for quality and pre-processed using MRtrix/3.0.2 software and FMRIB library in FSL/6.0.3 (https://mrtrix.readthedocs.io/, www.fmrib.ox.ac.uk/fsl/). Diffusion images were de-noised, corrected for Gibbs ringing artifact, head motion, eddy current distortions, and bias-field inhomogeneities. An advanced new method, known as the high-definition brain extraction tool^22^ was used to extract the brain tissue and generate individual brain masks.

Individual fractional anisotropy (FA) maps were then computed by fitting diffusion to the data tensors using FMRIB Diffusion Toolbox within brain masks. Using Tract Based Spatial Statistics, all FA images were nonlinearly transformed to the target image (FMRIB58_FA) and subsequently affine transformed to standard MNI152 space^23^. Hippocampal white matter integrity, i.e., hippocampal FA, was measured in two regions of interest bilaterally the fimbria and the hippocampal portion of the cingulum bundle defined according to the Johns Hopkins University- international consortium for brain mapping, white-matter atlas as described elsewhere^7^.

*N-acetylaspartate*

Hippocampal N-acetylaspartate (NAA), a marker of neuronal viability, was acquired from the left hippocampus using a standard short-echo point resolved spectroscopy sequence. Spectroscopic data were quantified using LCModel (version 6.3, LCMODEL, Oakville, ON, Canada), via a combination of water-suppressed and unsuppressed spectra, to compute the absolute quantification of each metabolite. This was achieved by fitting the experimental spectrum with a group of basis sets, each of which is the spectrum of a specific metabolite or macromolecule. We excluded low-quality spectral data (2 out of 111 images) by (i) discarding images with signal-to-noise ratios < 12 or global linewidth (full width at half maximum) < 0.1 ppm; and (ii) using a Cramer–Rao lower bound criterion of 10% for NAA total to further reject low-quality spectra. Of the remaining spectra, the parameters used for this study provided robust signals across intervention groups, and no significant group differences for any quality control measure (*p* > 0.05), with an average signal-to-noise ratio of 21.60 (SD = 3.33), full width at half maximum of 0.08 p.p.m. (SD = 0.02) and Cramer–Rao lower bounds of 3.30 (SD = 0.96).The volume fractions of different tissue types were calculated by initially segmenting T1 images in the individual space using SPM8. Then, a mask of the spectroscopic voxel was reconstructed using the dimension, placement and angulation information from MRS header information, then co-registered to the T1 image and extracted the volume of grey matter, white matter and cerebrospinal fluid within the voxel using an in-house script^24^. The fraction of cerebrospinal fluid was used to correct for the partial volume effect.

***Secondary Outcome Measures***

Key demographic characteristics, including age, gender, years of education, and age of regular cannabis use, were collected at baseline. Secondary outcomes assessing cannabis use, cognition, and mental health were assessed at baseline and endpoint.

***Substance Use***

The Timeline Follow Back Procedure (TLFB^25^) was used to determine the amount (grams) of cannabis consumed during the three months prior to baseline and throughout the intervention. The amount of cannabis consumed (grams) during the four weeks prior to baseline and endpoint assessment were the main TLFB outcome. The Severity of Dependence Scale (SDS^26^), and an adapted version of the Penn Alcohol Craving Scale (Penn Cannabis Craving score (PCCS^27^) were used to further characterise cannabis use. THC metabolites were determined from urine toxicology. Alcohol and nicotine use and dependence were assessed using the Alcohol Use Disorders Identification Test (AUDIT^28^) and Fagerström Test for Nicotine Dependence (FTND^29^), respectively.

***Mental Health and Cognition***

A battery of validated mental health and cognitive assessments were administered at baseline and endpoint including: The Quick Inventory of Depressive Symptomology (QIDS), The State Trait Anxiety Inventory-State (STAI-S), the Apathy Evaluation Scale (AES), the Connor-Davidson Resilience Scale – short form (CD-RISC-SF), the Warwick-Edinburgh Mental Wellbeing Scale - short form (WEMWBS-SF), the Quality of Life Enjoyment and Satisfaction Questionnaire – Short Form (Q-LES-Q-SF), and the Pittsburgh Sleep Quality Index (PSQI). Visual paired-associate learning was assessed using the CANTAB Paired Associate Learning test (PAL; Mean Error to Success (METS; average errors to successfully complete each stage), Total Errors Adjusted (TEA; total errors plus an adjustment for the estimated number of errors they would have made on any trials not completed), and First Attempts Memory Score (FAMS; number of times a participant chose the correct box on their first attempt when recalling the pattern locations). The Rey Auditory Verbal Learning Test (RAVLT Form A-C; Learning Score (A5-A1), Recall (A7) and Recognition) was used to assess verbal learning and memory. Alternate versions of each cognitive task were administered at endpoint.

***Task-based fMRI***

The Figural memory task (FigMem) see supplementary materials for details) was completed during fMRI using previously published methods^30^. The task began with a memory encoding phase where 20 abstract images were presented passively for participants to remember. A memory retrieving phase was then conducted after the DTI sequence (10 minutes). During memory retrieval, 40 images (including the 20 images from memory encoding and 20 new images) were presented, and participants were asked to decide whether the image was presented during memory encoding using a two-key button box with their right hand.

***Physical Activity***

The CARDIA physical activity history questionnaire^31^ was adapted and administered at baseline to assess typical activity levels over the previous 12- months and repeated at endpoint to capture physical activity levels throughout trial participation (i.e., in addition to HIIT and S&R sessions). As the original CARDIA physical activity history questionnaire captures 12-months of activity, a shortened version was created for the endpoint assessments to cover the intervention period. The language was also modified to ensure applicability in an Australian context. The primary outcome from the CARDIA was average MET-minutes of physical activity levels per week. To quantify total MET-minutes throughout the intervention, and determine change in exercise levels, endpoint MET-minutes for exercise outside the intervention were added to the MET-minutes engaged in during the intervention. This was calculated by multiplying exercise attendance (i.e., number of sessions) by the target MET-minutes per intervention session (225 MET-minutes), divided by 12 to obtain a weekly average.

***Cardiopulmonary Exercise Testing (CPET)***

The CPET protocol involved a continuous incremental running test on a treadmill (H/P/Cosmos Quasar Med 3P) to the point of volitional exhaustion. With a 9% fixed incline gradient, participants began the task at a metabolic equivalent (METs) of 4.6, increasing by 2.7 METs every 3 minutes. End-test criteria required participants to subjectively indicate to stop the test, signifying that they had reached volitional exhaustion. Participants wore a Polar H10 monitor which allowed calculation of their peak HR. Participants also wore a face mask to allow inhaled and expired gas to be analysed every 10 seconds using an online breath-by-breath system (ADInstruments). Inhaled and exhaled gas was measured to obtain a Respiratory Exchange Ratio (RER), which assesses the rate at which oxygen is consumed and carbon dioxide is produced^32^. An RER of 1.00 equates to an approximate lactate threshold. Participants were also asked to rate their perceived level of exertion every 60 seconds, using the Borg Scale. The Borg Scale asked the participants to point to a number from 6-20, with 6 being ‘no exertion’ and 20 being ‘maximal exertion’^33^. The rate of oxygen uptake at peak heart rate was recorded as V̇o_2max_.

***Exercise Engagement and Intensity***

Trial retention and exercise session attendance rates were assessed as the percentage of participants who completed the endpoint assessment and the percentage of exercise sessions attended, respectively. HR data was used to ascertain the amount of time participants exercised within set HR zones (above 70, 80 and 90% HRmax) and above their LT. LT was calculated as time above their heart rate at 1.00 RER, ascertained at baseline CPET.

**eResults**

***Participant Characteristics***

Fifty-nine, predominantly male (80%) participants, aged 20-53 (*M* = 27.00, *SD* = 6.26) completed baseline assessment. The majority of participants (76%) had severe CUD according to the MINI. The mean age of regular cannabis consumption was 19.52 (± 4.72) years, corresponding to 7.48 (± 6.49) years of regular cannabis use.

eTable 4. *Demographic and Baseline Characteristics*

|  | Overall | HIIT | S&R |
| --- | --- | --- | --- |
| Demographics | | | |
| Age (years) | 27.0 (6.26) | 26.52 (5.72) | 27.47 (6.80) |
| Gender (male/female) | 47/12 | 22/7 | 25/5 |
| Engagement | | | |
| Retention (%) | 79.67 | 79.67 | 80% |
| Attendance (%) | 79.51 (30.01) | 77.97 (31.34) | 81.02 (29.26) |
| Baseline cannabis use measures | | | |
| Dependence (SDS) | 5.10 (2.81) | 5.56 (2.98) | 4.67 (2.62) |
| Cannabis craving (PCCS) | 15.02 (5.29) | 15.07 (6.52) | 14.97 (3.87) |
| MINI severity (moderate/severe) | 13/46 | 6/23 | 7/23 |
| Frequency (days per month) | 23.85 (8.30) | 23.93 (8.09) | 23.77 (8.64) |
| Past 3 months usage (grams) | 123.99 (125.83) | 131.07 (132.48) | 116.90 (120.72) |
| Age of regular use (years) | 19.52 (4.72) | 18.96 (4.90) | 20.06 (4.56) |
| Years of use | 7.48 (6.49) | 7.56 (7.56) | 7.40 (5.38) |
| THC levels in urine (ug/L) | 321.15 (315.38) | 337.74 (286.19) | 310.28 (337.63) |
| Baseline substance use measures | | | |
| Alcohol dependence score (AUDIT dependence score) | 0.39 (0.79) | 0.42 (0.98) | 0.37 (0.56) |
| Alcohol consumption score (AUDIT consumption score) | 4.09 (2.34) | 3.83 (2.09) | 4.33 (2.58) |
| AUDIT total score | 5.66 (4.26) | 5.79 (4.73) | 5.53 (3.52) |
| Nicotine dependence (FTND) | 1.05 (1.72) | 0.97 (1.78) | 1.13 (1.68) |
| Baseline mental health measures | | | |
| Depression (QIDS) | 7.50 (4.76) | 7.64 (4.65) | 7.37 (4.93) |
| State anxiety (STAI-S) | 32.57 (8.60) | 33.75 (8.49) | 31.47 (8.62) |
| Apathy (AES) | 58.40 (7.17) | 59.86 (6.74) | 57.03 (7.41) |
| Resilience (CD_RISC-SF) | 29.57 (6.41) | 29.57 (6.82) | 29.57 (6.12) |
| Wellbeing (WEMWBS-SF) | 25.31 (4.57) | 25.50 (4.51) | 25.13 (5.41) |
| Quality of Life (Q-LES-Q-SF) | 52.36 (8.55) | 52.50 (7.63) | 52.23 (9.45) |
| Sleep (PSQI) | 6.25 (2.80) | 5.6071 (2.60) | 6.8929 (2.90) |
| Baseline hippocampal integrity | | | |
| Hippocampal Integrity Index | 0.08 (1.97) | -0.26 (2.13) | 0.37 (1.83) |
| Hippocampal Volume | 4046.12 (385.73) | 4034.72 (415.09) | 4057.93 (360.05) |
| NAA concentration | 5.50 (0.63) | 5.36 (0.60) | 5.63 (0.64) |
| Hippocampal FA | 0.28 (0.03) | 0.29 (0.02) | 0.28 (0.02) |
| Baseline cognitive measures | | | |
| RAVLT - Learning score | 5.59 (2.46) | 6.07 (2.51) | 5.13 (2.36) |
| RAVLT - Recall | 9.97 (3.11) | 10.66 (3.05) | 9.30 (3.08) |
| RAVLT - Recognition | 47.08 (2.97) | 47.86 (2.45) | 46.33 (3.27) |
| PAL - Mean Errors to Success | 1.08 (1.09) | 1.14 (1.06) | 1.03 (1.13) |
| PAL - Total Errors Adjusted | 6.66 (5.95) | 7.17 (6.14) | 6.17 (5.83) |
| PAL – First Attempt Memory Score | 15.39 (3.50) | 15.21 (3.29) | 15.57 (3.75) |
| Baseline exercise | | | |
| V̇o_2max_ | 38.85 (7.66) | 37.80 (7.49) | 39.87 (7.80) |
| Average physical activity (METS per week) | 916.88 (908.22) | 1039.30 (999.11) | 811 (860.58) |

NB: SDS, Severity of dependence scale; PCCS, Penn cannabis craving scale; MINI, Mini International Neuropsychiatric Interview; Timeline Follow-back Procedures used to ascertain 3-month dosage and frequency of days; QIDS, Quick Inventory of Depressive Symptomatology; STAI, State and Trait Anxiety Inventory; AES, Apathy Evaluation Scale; CD-RISC-SF, Connor-Davidson Resilience Scale - short form; WEMWBS-SF, Warwick-Edinburgh Mental Wellbeing Scale - short form; Q-LES-Q-SF, Quality of Life Enjoyment and Satisfaction Questionnaire – Short Form; PSQI, Pittsburgh Sleep Quality Index (PSQI); RAVLT, Rey Auditory Verbal Learning Test; PAL, Paired Associate Learning.

^a^ Mann Whitney U-tests

^b^ χ2-test

***Participant Retention and Adherence***

Overall, 47 of the 59 participants completed the 12-week intervention (79.67%). Two participants withdrew after baseline and did not receive the intervention. Ten withdrew throughout the intervention: due to competing life demands (*n* = 6), the commencement of an excluded medication (*n* = 1), and the COVID-19 pandemic (*n* = 3). On average, participants attended 80% of the prescribed exercise sessions and this rate did not differ between groups (*p* > 0.05). The HIIT group spent significantly more time exercising above 70%- (*U* = 231.5, *z* = -2.79, *p* < 0.01), 80%- (*U* = 121.5, *z* = -4.54, *p* < 0.001) and 90%-HRmax (*U* = 88.5, *z* = -5.10, *p* < 0.001), and lactate threshold (*U* = 172.0, *z* = -3.74, *p* < 0.001), in comparison to the S&R group. Exercise attendance significantly increased following the COVID-19 pandemic and subsequent introduction of video based sessions, however average time per session spent exercising in each HR zone and above LT did not differ in participants who had sessions via video-call (see supplementary material, eTable 5). Average exercise engagement (weekly MET-minutes) increased significantly from baseline to endpoint, regardless of condition (main effect of time, Wald χ^2^ = 5.199, *p* = 0.02). V̇o_2max_ did not increase significantly following the HIIT or S&R intervention in the intention to treat analysis (*p* = 0.09), however a significant time *(p < 0.01)* and time by group *(p < 0.01)* interaction were revealed in the per protocol analysis with the increase in V̇o_2max_ being greater for the HIIT group.

eTable 5. Exercise engagement between participants who did, or did not have at least one exercise session via video-call.

|  | ***Had sessions via video-call*** | | ***Had no sessions via video-call*** | |  |
| --- | --- | --- | --- | --- | --- |
|  | ***M*** | ***SD*** | ***M*** | ***SD*** | ***p*** |
| ***Exercise attendance (number of sessions)*** | | | | | |
|  | *32.89* | *7.34* | *25.03* | *12.04* | <0.01 |
| ***Exercise intensity (hours per session)*** | | | | | |
| **Above LT** | 0.14 | 0.12 | 0.11 | 0.11 | 0.33 |
| **Above 90% HRmax** | 0.03 | 0.04 | 0.04 | 0.05 | 0.53 |
| **Above 80% HRmax** | 0.12 | 0.09 | 0.12 | 0.10 | 0.94 |
| **Above 70% HRmax** | 0.27 | 0.12 | 0.25 | 0.12 | 0.50 |

**eReferences**

1.	Firth J, Rosenbaum S, Stubbs B, Gorczynski P, Yung AR, Vancampfort D. Motivating factors and barriers towards exercise in severe mental illness: a systematic review and meta-analysis. *Psychol Med*. 2016;46(14):2869-2881. doi:10.1017/S0033291716001732

2.	Türkmen C, Martland R, Grilli M, Stubbs B, Roessler KK, Hallgren M. Can high-intensity interval training improve health outcomes among people with substance use disorders? A systematic review and preliminary meta-analysis. *Ment Health Phys Act*. 2024;27:100622. doi:10.1016/j.mhpa.2024.100622

3.	Schuch FB, Vancampfort D, Richards J, Rosenbaum S, Ward PB, Stubbs B. Exercise as a treatment for depression: A meta-analysis adjusting for publication bias. *J Psychiatr Res*. 2016;77:42-51. doi:10.1016/j.jpsychires.2016.02.023

4.	Jacka FN, O’Neil A, Opie R, et al. A randomised controlled trial of dietary improvement for adults with major depression (the ‘SMILES’ trial). *BMC Med*. 2017;15(1):23. doi:10.1186/s12916-017-0791-y

5.	The Diabetes Prevention Program (DPP) Research Group. The Diabetes Prevention Program (DPP). *Diabetes Care*. 2002;25(12):2165-2171. doi:10.2337/diacare.25.12.2165

6.	Boutron I, Altman DG, Moher D, Schulz KF, Ravaud P, CONSORT NPT Group. CONSORT statement for randomized trials of nonpharmacologic treatments: A 2017 update and a CONSORT extension for nonpharmacologic trial abstracts. *Ann Intern Med*. 2017;167(1):40-47. doi:10.7326/M17-0046

7.	Yücel M, Lorenzetti V, Suo C, et al. Hippocampal harms, protection and recovery following regular cannabis use. *Transl Psychiatry*. 2016;6(1):e710-e710. doi:10.1038/tp.2015.201

8.	Chye Y, Lorenzetti V, Suo C, et al. Alteration to hippocampal volume and shape confined to cannabis dependence: a multi‐site study. *Addict Biol*. 2019;24(4):822-834. doi:10.1111/adb.12652

9.	Yücel M, Solowij N, Respondek C, et al. Regional brain abnormalities associated with long-term heavy cannabis use. *Arch Gen Psychiatry*. 2008;65(6):694-701. doi:doi: 10.1001/archpsyc.65.6.694

10.	Owens MM, Sweet LH, MacKillop J. Recent cannabis use is associated with smaller hippocampus volume: High‐resolution segmentation of structural subfields in a large non‐clinical sample. *Addict Biol*. 2021;26(1):e12874. doi:10.1111/adb.12874

11.	Meier MH, Caspi A, R. Knodt A, et al. Long-term cannabis use and cognitive reserves and hippocampal volume in midlife. *Am J Psychiatry*. 2022;179(5):362-374. doi:10.1176/appi.ajp.2021.21060664

12.	Zalesky A, Solowij N, Yucel M, et al. Effect of long-term cannabis use on axonal fibre connectivity. *Brain*. 2012;135(7):2245-2255. doi:10.1093/brain/aws136

13.	Solowij N. Chapter 40 - Chronic Cannabis Use and Axonal Fiber Connectivity.

14.	Jager G, Van Hell HH, De Win MML, et al. Effects of frequent cannabis use on hippocampal activity during an associative memory task. *Eur Neuropsychopharmacol*. 2007;17(4):289-297. doi:10.1016/j.euroneuro.2006.10.003

15.	Campbell S, Macqueen G. The role of the hippocampus in the pathophysiology of major depression. *J Psychiatry Neurosci JPN*. 2004;29(6):417-426.

16.	Videbech P, Ravnkilde B. Hippocampal volume and depression: a meta-analysis of MRI studies. *Am J Psychiatry*. 2004;161(11):1957-1966. doi:10.1176/appi.ajp.161.11.1957

17.	Ghasemi M, Navidhamidi M, Rezaei F, Azizikia A, Mehranfard N. Anxiety and hippocampal neuronal activity: Relationship and potential mechanisms. *Cogn Affect Behav Neurosci*. 2022;22(3):431-449. doi:10.3758/s13415-021-00973-y

18.	Pimontel MA, Kanellopoulos D, Gunning FM. Neuroanatomical abnormalities in older depressed adults with apathy: A systematic review. *J Geriatr Psychiatry Neurol*. 2020;33(5):289-303. doi:10.1177/0891988719882100

19.	Zeng N, Aleman A, Liao C, Fang H, Xu P, Luo Y. Role of the amygdala in disrupted integration and effective connectivity of cortico-subcortical networks in apathy. *Cereb Cortex*. 2023;33(6):3171-3180. doi:10.1093/cercor/bhac267

20.	Patenaude B, Smith SM, Kennedy DN, Jenkinson M. A Bayesian model of shape and appearance for subcortical brain segmentation. *NeuroImage*. 2011;56(3):907-922. doi:10.1016/j.neuroimage.2011.02.046

21.	Thompson PM, Jahanshad N, Ching CRK, et al. ENIGMA and global neuroscience: A decade of large-scale studies of the brain in health and disease across more than 40 countries. *Transl Psychiatry*. 2020;10(1):100. doi:10.1038/s41398-020-0705-1

22.	Isensee F, Schell M, Pflueger I, et al. Automated brain extraction of multisequence MRI using artificial neural networks. *Hum Brain Mapp*. 2019;40(17):4952-4964. doi:10.1002/hbm.24750

23.	Smith SM, Jenkinson M, Johansen-Berg H, et al. Tract-based spatial statistics: Voxelwise analysis of multi-subject diffusion data. *NeuroImage*. 2006;31(4):1487-1505. doi:10.1016/j.neuroimage.2006.02.024

24.	Kirkovski M, Suo C, Enticott PG, Yücel M, Fitzgerald PB. Short communication: Sex-linked differences in gamma-aminobutyric acid (GABA) are related to social functioning in autism spectrum disorder. *Psychiatry Res Neuroimaging*. 2018;274:19-22. doi:10.1016/j.pscychresns.2018.02.004

25.	Robinson SM, Sobell LC, Sobell MB, Leo GI. Reliability of the Timeline Followback for cocaine, cannabis, and cigarette use. *Psychol Addict Behav*. 2014;28(1):154-162. doi:10.1037/a0030992

26.	Van Der Pol P, Liebregts N, De Graaf R, Korf DJ, Van Den Brink W, Van Laar M. Reliability and validity of the Severity of Dependence Scale for detecting cannabis dependence in frequent cannabis users. *Int J Methods Psychiatr Res*. 2013;22(2):138-143. doi:10.1002/mpr.1385

27.	Flannery BA, Volpicelli JR, Pettinati HM. Psychometric properties of the penn alcohol craving scale. *Alcohol Clin Exp Res*. 1999;23(8):1289-1295. doi:10.1111/j.1530-0277.1999.tb04349.x

28.	Saunders JB, Aasland OG, Babor TF, De La Fuente JR, Grant M. Development of the Alcohol Use Disorders Identification Test (AUDIT): WHO Collaborative Project on Early Detection of Persons with Harmful Alcohol Consumption‐II. *Addiction*. 1993;88(6):791-804. doi:10.1111/j.1360-0443.1993.tb02093.x

29.	Heatherton TF, Kozlowski LT, Frecker RC, Fagerstrom K. The Fagerström Test for Nicotine Dependence: a revision of the Fagerstrom Tolerance Questionnaire. *Br J Addict*. 1991;86(9):1119-1127. doi:10.1111/j.1360-0443.1991.tb01879.x

30.	Jamadar S, Assaf M, Jagannathan K, Anderson K, Pearlson GD. Figural memory performance and functional magnetic resonance imaging activity across the adult lifespan. *Neurobiol Aging*. 2013;34(1):110-127. doi:10.1016/j.neurobiolaging.2012.07.013

31.	Jacobs DR, Hahn LP, Haskell WL, Pirie P, Sidney S. Validity and reliability of short physical activity history: cardia and the Minnesota Heart Health Program. *J Cardiopulm Rehabil Prev*. 1989;9(11):448-459.

32.	Solberg G, Robstad B, Skjønsberg OH, Borchsenius F. Respiratory gas exchange indices for estimating the anaerobic threshold. *J Sports Sci Med*. 2005;4(1):29-36.

33.	Williams N. The borg rating of perceived exertion (RPE) scale. *Occup Med*. 2017;67(5):404-405. doi:10.1093/occmed/kqx063

34.	Zeger SL, Liang KY, Albert PS. Models for longitudinal data: a generalized estimating equation approach. *Biometrics*. 1988;44(4):1049. doi:10.2307/2531734
